# Novel Transgastric Endoluminal Segmental Esophagectomy and Primary Anastomosis Technique: A Hybrid Transgastric Thoracoscopic Esophagectomy for the Treatment of High Grade Dysplasia and Early Esophageal Cancer in a Porcine *Ex vivo* Model

**DOI:** 10.3389/fsurg.2021.676031

**Published:** 2021-07-01

**Authors:** Anton Kvasha, Muhammad Khalifa, Seema Biswas, Moaad Farraj, Zakhar Bramnik, Igor Waksman

**Affiliations:** ^1^Ziv Medical Center, Safed, Israel; ^2^The Azrieli Faculty of Medicine, Bar-Ilan University, Tzfat, Israel; ^3^Galilee Medical Center, Nahariya, Israel; ^4^The Baruch Padeh Medical Center, Tiberias, Israel

**Keywords:** endolumenal, esophagecotmy, thorascopic surgery, high grade dysplasia, esophageal cancer

## Abstract

Multiple modalities are currently employed in the treatment of high grade dysplasia and early esophageal carcinoma. While they are the subject of ongoing investigation, surgery remains the definitive modality for oncological resection. Esophagectomy, however, is traditionally a challenging surgical procedure and carries a significant incidence of morbidity and mortality. Endoscopic mucosal resection (EMR) and endoscopic submucosal dissection (ESD) are considerably less invasive alternatives to esophagectomy in the diagnosis and treatment of high grade dysplasia, early esophageal squamous cell carcinoma and adenocarcinoma. However, many early esophageal cancer patients, with favorable histology, who could benefit from endoscopic resection, are referred for formal esophagectomy due to lesion characteristics such as unfavorable lesion morphology or recurrence after previous endoscopic resection. In this study we present a novel, hybrid thoracoscopic transgastric endoluminal segmental esophagectomy with primary anastomosis for the potential treatment of high grade dysplasia and early esophageal cancer in a porcine *ex vivo* model as a proposed bridge between endoscopic resection and the relatively high mortality and morbidity formal esophagectomy procedure. The novel technique consists of thoracoscopic esophageal mobilization in addition to transgastric endoluminal segmental esophagectomy and anastomosis utilizing a standard circular stapler. The technique was found feasible in all experimental subjects. The minimally invasive nature of this novel procedure as well as the utility of basic surgical equipment and surgical skill is an important attribute of this method and can potentially make it a treatment option for many patients who would otherwise be referred for a formal esophagectomy.

## Introduction

Mucosal ablation, resection and cryotherapy are currently employed in the treatment of high grade dysplasia and early esophageal carcinoma. While their indication, safety and effectiveness are the subject of ongoing investigation and debate, surgery remains the definitive modality for oncological resection especially for intramucosal and submucosal lesions where the risk of nodal spread is a significant threat (1). Esophagectomy, however, is traditionally a challenging surgical procedure and carries a significant incidence of morbidity and mortality even when this is reduced to 8% in high volume centers (2). Numerous attempts have been made over time to reduce the morbidity and mortality rates of esophagectomy procedures. Much of this effort was directed at reducing the inherent invasiveness of the procedure which may involve operating in the thoracic, abdominal, and cervical compartments. Minimally invasive esophagectomy (MIE) for the management of esophageal cancer was first described by Cuschieri et al. (3) in 1992. This procedure involved thoracoscopic esophageal mobilization, and gastric mobilization by laparotomy; this is still a common approach to esophagectomy today (4). Later, the thoracoscopic technique was modified by Luketich et al. (5), who described a thoracolaparoscopic esophagectomy. Totally laparoscopic transhiatal esophagectomy was first described by DePaula et al. (6) in 1995 and a variety of MIE procedures exist today including robotically assisted THE (7, 8).

In addition to thoracoscopy and laparoscopy, endoscopy is another minimally invasive approach utilized in the treatment of esophageal disease. Endoscopic mucosal resection (EMR) and endoscopic submucosal dissection (ESD) are considerably less invasive alternatives to esophagectomy in the diagnosis and treatment of high grade dysplasia (HGD), early esophageal squamous cell carcinoma (SCC), and adenocarcinoma. When esophagectomy is compared with endoscopic therapy for the treatment of early-stage (T1a) esophageal cancer, similar 5 year survival is found, however, cancer free survival and recurrence rate results favor the esophagectomy group (9). Albeit its minimally invasive nature, complications of EMR include bleeding, perforation, fibrosis and stricture formation at the site of resection. The most common of these is stricture formation (10). In fact, EMR is not recommended for circumferential lesions due to the relatively high incidence of strictures (11). Other lesion characteristics, including, but not limited to, large and non-lifting lesions render EMR unsuitable and may serve as an indication for ESD. At present ESD is not widely available in western countries and its superiority needs further support.

Currently, early esophageal cancer lesions deemed unsuitable for endoscopic resection (EMR or ESD) are referred for formal esophagectomy. Unfortunately, at present there are no procedures which bridge the endoscopic and surgical approaches combining the low morbidity of endoscopic treatment and the radical nature of formal, open or minimally invasive, esophagectomy. To address this problem, we present a novel, hybrid thoracoscopic, transgastric endoluminal segmental esophagectomy with primary anastomosis for the potential treatment of high grade dysplasia and early esophageal cancer in a porcine *ex vivo* model.

## Materials and Methods

**Figure 1:**
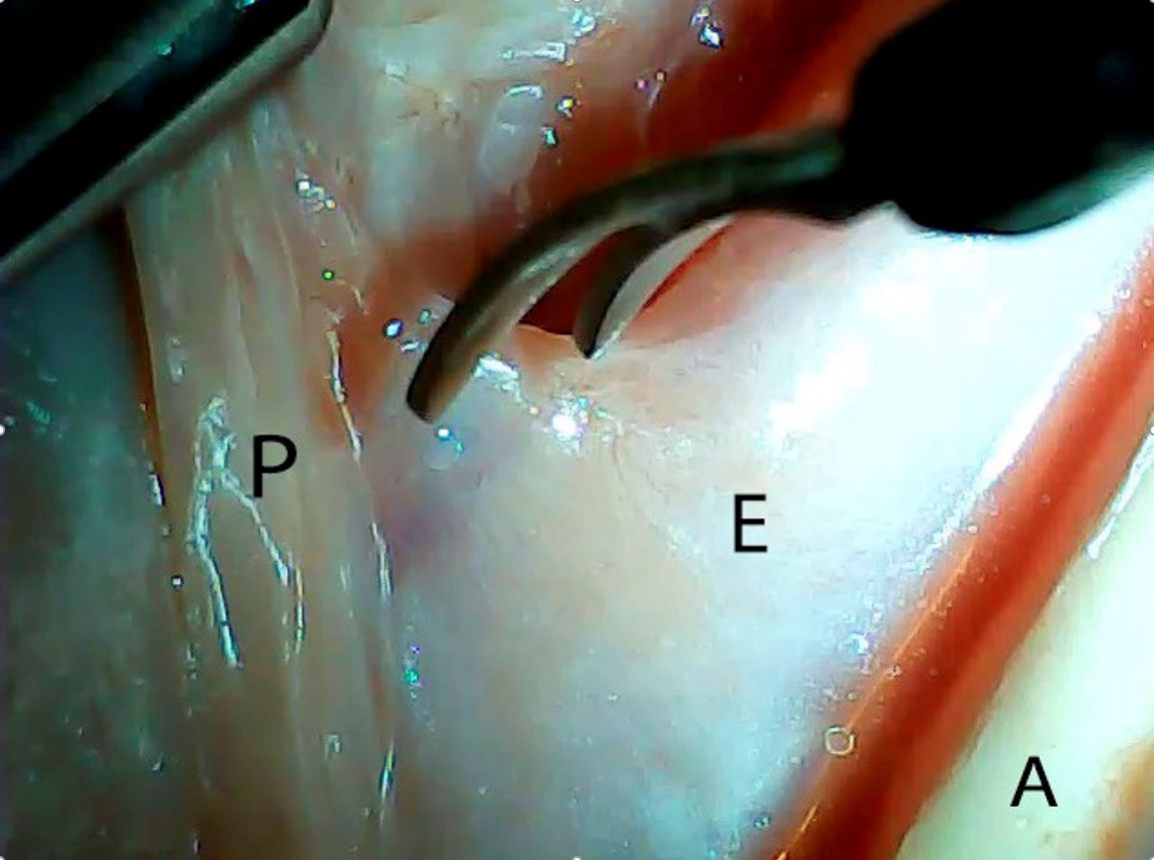
Thoracoscopic mobilization of the middle third of porcine esophagus. P, parietal pleura; E, esophagus; A, aorta.

**Figure 2:**
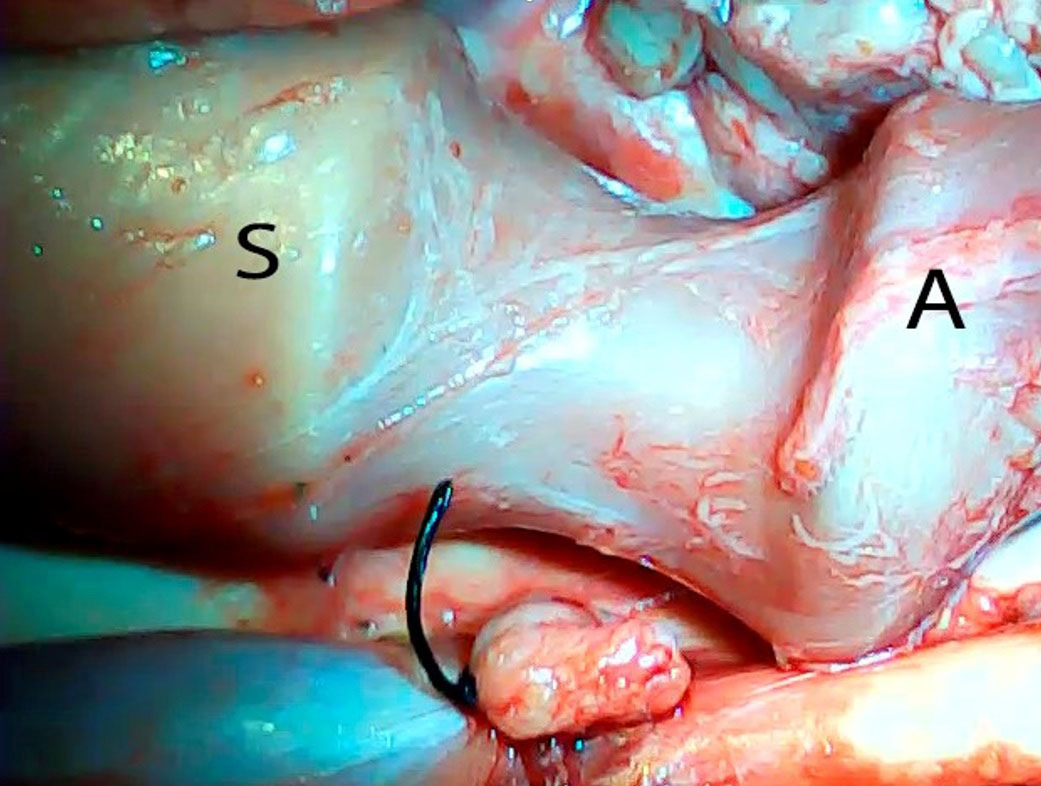
Opening of the circular stapler within the mobilized portion of the esophagus. S, stapler; A, anvil.

**Figure 3:**
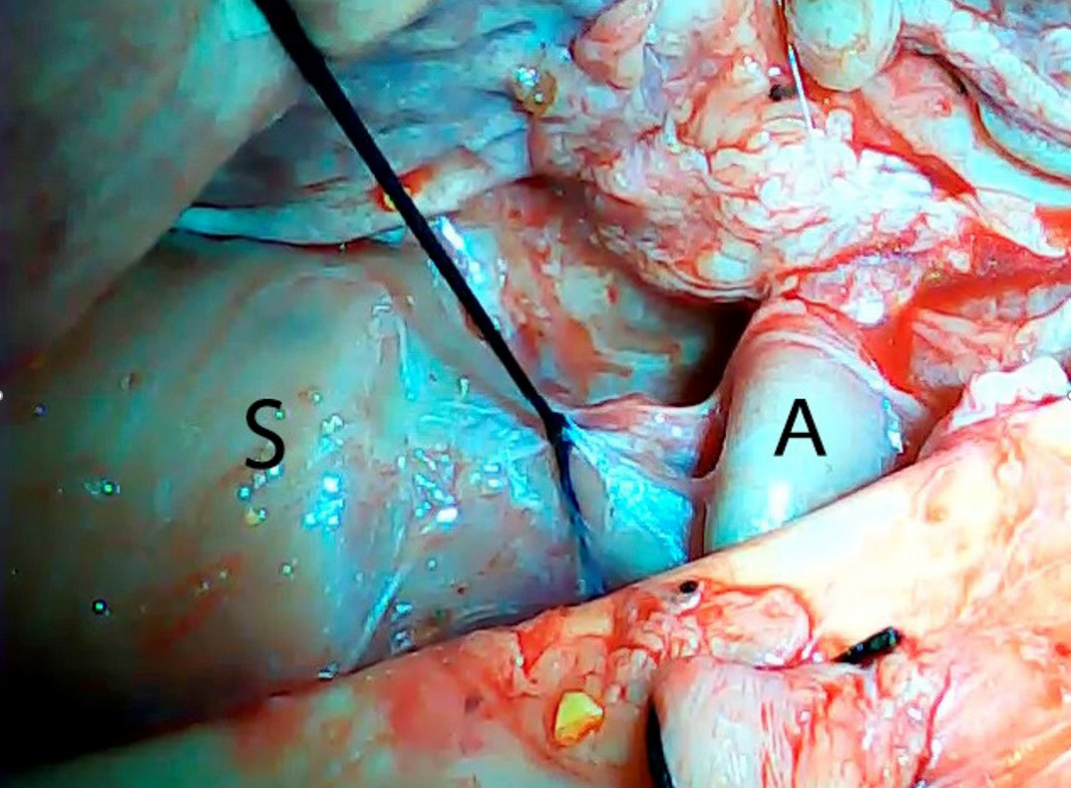
Thoracoscopic silk tie around the mobilized esophagus attaching it to the shaft of the anvil. S, stapler; A, anvil.

**Figure 4:**
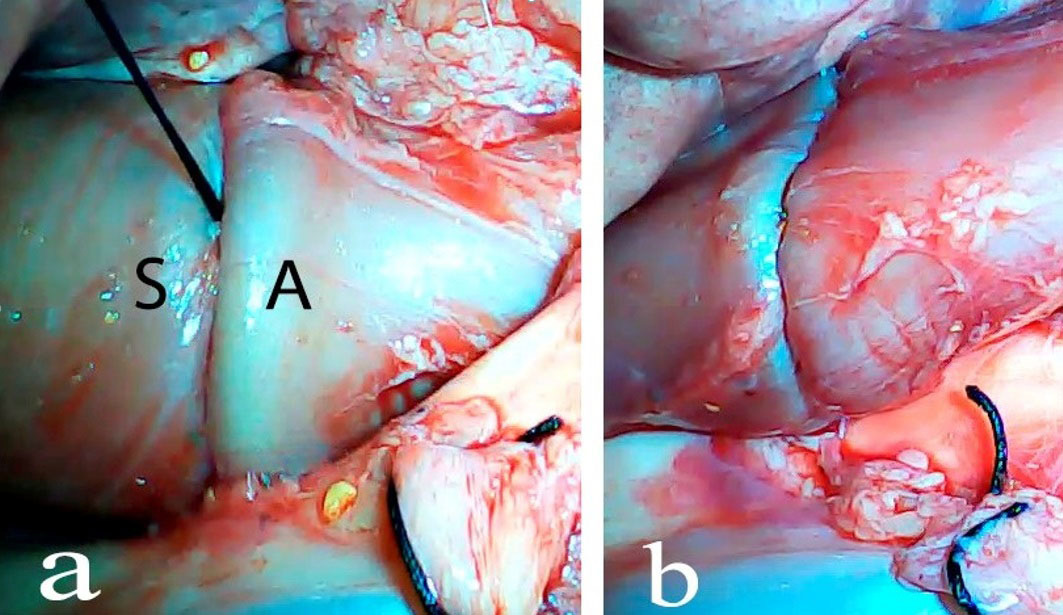
Closure of the stapler and stapler deployment. S, stapler; A, anvil **(a)**, esophago-esophageal anastomosis after stapler deployment and transgastric stapler extraction **(b)**.

**Figure 5:**
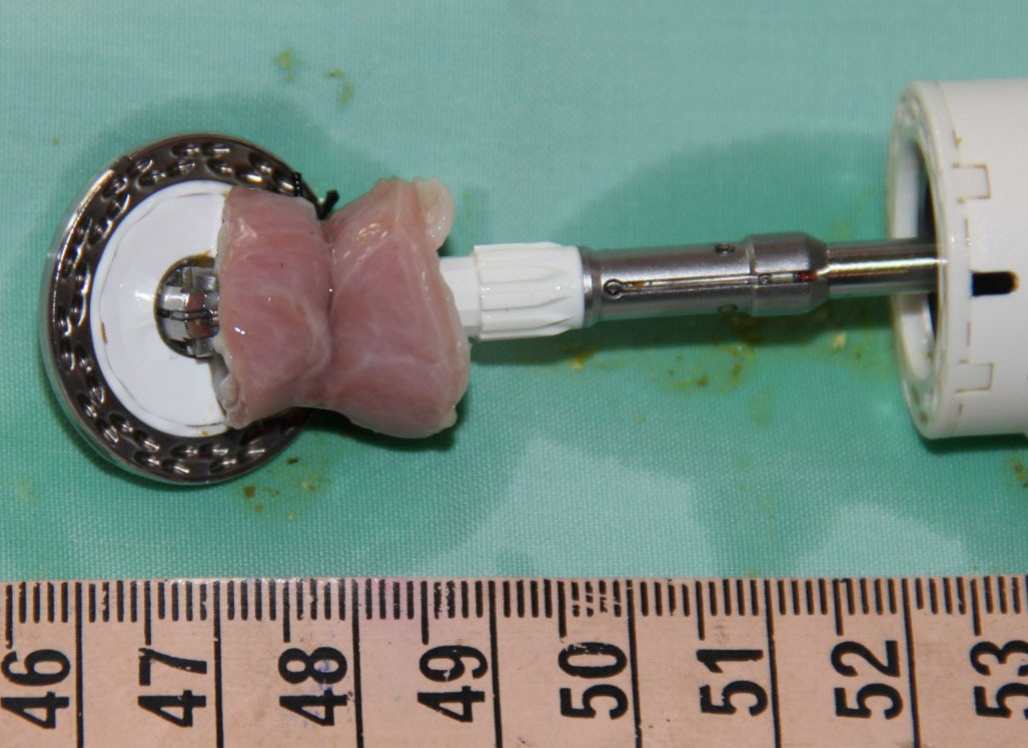
Full thickness esophageal segment resected utilizing transgastric endolumenal segmental esophagectomy technique.

An *ex vivo*, feasibility study was performed utilizing 5 female (Sus scrofa domestica) pigs with an average weight of 68 kg. The study and use of pigs as an animal model was approved by the institutional veterinarian. We performed a left thoracoscopy because in a porcine model it provides the best access to middle esophagus. Each of the sacrificed animals was placed in the right lateral decubitus position. A 5 mm thoracoscopic camera was inserted 10 cm cephalad to the left costal margin. A 5 mm trocar was inserted in an intercostal space 5 cm cephalad to the camera. Another 5 mm trocar was placed in the second intercostal space caudad to the camera. Eight to twelve centimeters of middle thoracic esophagus was mobilized utilizing laparoscopic scissors (Figure 1). Once the esophagus was mobilized, a small horizontal, lateral abdominal incision was performed 5 cm caudal to the left costal margin. The stomach was pulled through the incision and a 3 cm gastrotomy was performed. An end-to-end circular stapler (Autosuture, EEA™ 25 mm single-use stapler, Covidien, CT, USA) connected extracorporeally to its anvil, was passed into the esophagus through the gastrotomy in a closed orientation. The stapler was then opened intralumenally in the middle of the mobilized segment of the esophagus (Figure 2). A silk suture ligation was performed thoracoscopically around the esophagus attaching the esophagus to the shaft of the anvil (Figure 3). An endoluminal segmental esophageal resection and anastomosis was performed by deploying the EEA stapler (Figure 4). The stapler was extracted through the gastrotomy and the resected esophagus examined (Figure 5).

## Results

All five pigs successfully underwent the hybrid thoracoscopic transgastric endoluminal segmental esophagectomy. Transgastric insertion of a 25 mm EEA stapler into the esophagus was undertaken with no difficulty in all five experimental subjects. Intralumenal opening of the stapler was performed with no hindrance; no anvil dislocation was observed in any of the experimental subjects. The laparoscopically applied silk tie at the resection target site enabled a stable association between the esophagus and the stapler in all cases. Closure of the stapler over the esophagus tied to the shaft of the anvil established an appropriate configuration for segmental esophageal resection and anastomosis by deploying the EEA stapler. Transgastric extraction of the resected esophageal segment connected to the stapler was performed without any hindrance. The mean procedural time was 74 min (Table 1). The macroscopic analysis of the specimens from all experimental subjects revealed the presence of an intact thoracoscopic suture, which served as a marker of a full thickness resection. The resected esophageal segments averaged 2.1 cm (Table 1). Macroscopic analysis showed standard features of an end-to-end anastomosis created by a circular stapler. No esophageal tension was observed after the segmental resection.

**Table 1:** Five experimental subjects undergone endoluminal transgastric esophagectomies.

**Experiment**	**Length (cm)**	**Time (min)**	**Esophageal resection**
1	2.3	90	Mid esophagus
2	1.8	86	Mid esophagus
3	1.9	75	Mid esophagus
4	2.4	58	Mid esophagus
5	2.3	62	Mid esophagus
Average	2.1	74	
Range	0.6	32	

*Lengths of segmental esophageal resection and procedural times are listed in centimeters and minutes, respectively*.

## Discussion

Mortality from esophageal cancer remains high in spite of advances in medical therapy as the incidence of SCC of the esophagus remains unchanged, and the incidence of the esophageal adenocarcinoma continues to increase (12). The traditional, open, total, and distal esophagectomy procedures have become less invasive through the introduction of video assisted thoracoscopic surgery (VATS) and totally thoracoscopic-laparoscopic procedures in esophageal dissection and reconstruction. Commonly utilized minimally invasive esophagectomy today (VATS/laparotomy) still carries a 30-day mortality of 3.3% (13). Further advances in the field of minimally invasive esophagectomy include totally thoracolaparoscopic esophagectomy, totally laparoscopic transhiatal esophagectomy as well as robotically assisted procedures. However, these procedures are technically challenging and a minimum of 34 thoracoscopic esophagectomies are needed to accomplish a difference in outcome according to Osugi et al. (14) when performing a VATS esophagectomy combined with laparotomy. In spite of the efforts made in innovative design, training and proficiency in these procedures, the benefits of minimally invasive esophagectomy remain unclear. In fact, a 6 year randomized Phase III study was commenced in May 2015 in Japan to compare thoracoscopic esophagectomy to open esophagectomy (15).

Endoscopic mucosal resection (EMR) and endoscopic submucosal dissection (ESD) are alternatives to esophagectomy in the staging, grading and treatment of high grade dysplasia (HGD), early esophageal SCC and adenocarcinoma. EMR is a treatment option for low risk, early esophageal carcinoma lesions defined by macroscopically polypoid or flat lesions, with a histological pattern of sm1 invasion, good-to-moderate differentiation [G1/2], and no invasion into lymph vessels or veins (16). However, limitations of EMR include lesion size and no more than 2/3 of esophageal circumference (17, 18). Further, additional lesion characteristics which render these unsuitable for EMR are piecemeal resection (19), failure of mucosal lifting after injection, and post-endoscopic treatment recurrence (20). Finally, although endoscopic treatment of early esophageal cancer offers similar 5 year survival as surgical treatment, cancer free survival and recurrence favor the surgical group. Prasad et al. report recurrence rates of 2 and 12% for the surgical and endoscopic groups, respectively (9).

This study illustrates the feasibility of a novel, hybrid thoracoscopic, transgastric endoluminal segmental esophagectomy with primary anastomosis in an *ex vivo* porcine model. The procedure demonstrates successful and consistent segmental esophageal resection and anastomosis in a relatively short procedure time (Average 74 min) compared to a two or three field esophagectomy. The motivation behind this novel technique is to propose a bridge between EMR and the relatively high mortality and morbidity of formal esophagectomy since ESD is not widely available. Moreover, even if ESD availability were not an issue, the superiority of ESD over EMR is currently subject to debate (21, 22). The endoluminal segmental esophagectomy technique is designed to address high grade dysplasia and early esophageal carcinoma lesions not suitable for EMR, namely lesions of over 2/3 of esophageal circumference, non-lifting lesions, lesions which require piece meal resection or recurrent lesions. Moreover, endoluminal segmental esophagectomy, due to its technical simplicity and minimally invasive nature may be applied to any lesion suitable for EMR or ESD when endoscopic treatment is not available, therefore omitting a potentially unnecessary formal esophagectomy in the treatment of early esophageal carcinoma.

The utility of basic surgical equipment and the requirement of only basic surgical and thoracoscopic skills are important attributes of the endoluminal segmental esophagectomy technique. Transgastric insertion of the circular stapler in its closed orientation was accomplished without difficulty. The thoracoscopically placed suture established a stable association between the esophagus and the stapler to facilitate a “one-step” segmental esophagectomy and primary anastomosis utilizing a standard circular stapler. This may make the procedure more accessible and limit the complications associated with the learning curve typical of complex surgical and endoscopic procedures.

In addition to providing a hybrid minimally invasive, abbreviated, esophagectomy alternative to a formal, two or three field esophagectomy, the endoluminal segmental esophagectomy technique may be considered a clean, endoluminal esophagectomy procedure. The clean classification is appropriate for the thoracic cavity since esophageal resection margins face into the lumen of the esophagus as in the natural orifice specimen extraction (NOSE) endoluminal rectal intussusception and pull though (IPT) techniques (23). As previously shown, “clean contaminated” bowel resection may be associated with a contamination rate as high as 28% (24). Our group found no contamination of the abdominal cavity when the endoluminal IPT technique was utilized for rectal resection in three separate porcine model studies (25). Therefore, endoluminal segmental esophagectomy is expected to have similar thoracic cavity contamination rates as other endoluminal bowel resection techniques utilizing the same surgical principles.

This technique is designed for lesions not involving the GE junction, which may be a short coming for the treatment of esophageal adenocarcinoma predominant in developed countries. However, endoluminal segmental esophagectomy has the potential to become a viable treatment option for early SCC. Early SCC tends to involve the proximal and middle esophagus, and is the predominant subtype worldwide, comprising ~90% of all esophageal cancers, particularly in areas with the highest incidence of esophageal cancer, such as the “Asian Esophageal Cancer Belt” extending from North Iran to North-Central China, and into Russia (4). The simplicity and basic equipment requirements of the endoluminal segmental esophagectomy technique may be a significant advantage in these endemic areas.

There are several obvious limitations to this research so far. In the current study, our group did not attempt localization of the esophageal segment planned for resection and, therefore, did not address infiltrated margins. Furthermore, current research was conducted utilizing a small, *ex vivo*, animal feasibility model. We are planning to address these shortcomings by performing a similar study on a larger, survival, experimental subject group utilizing endoscopic simulation of malignancy targeted for esophageal segment localization, histological specimen analysis, laparoscopy for the abdominal part of the procedure, multiple-stapling and repeat endoluminal esophagectomy for infiltrated margins.

Although more investigation is warranted, the novel hybrid thoracoscopic endoluminal segmental esophagectomy procedure reported in this paper has the potential to omit an unnecessary two or three field esophagectomy for early esophageal carcinoma lesions which cannot be removed endoscopically either because of unfavorable lesion morphology or due to the lack of availability of advanced endoscopic services.

## Data Availability Statement

The original contributions presented in the study are included in the article/supplementary material, further inquiries can be directed to the corresponding author/s.

## Ethics Statement

The animal study was reviewed and approved by Dr. Weis Israel, Head of Veterinary Services, Galilee Medical Center, Nahariya, Israel.

## Author Contributions

MK: surgical procedure performer, study design, and article writing and editing. AK: surgical procedure design and development, study design, surgical procedure performer, and article writing and editing. SB, MF, ZB, and IW: article writing and editing. All authors contributed to the article and approved the submitted version.

## Conflict of Interest

The authors declare that the research was conducted in the absence of any commercial or financial relationships that could be construed as a potential conflict of interest.

## References

[1] Watson TJ. Esophagectomy for superficial esophageal neoplasia. Gastrointest Endosc Clin N Am. (2017) 27:531–54. 10.1016/j.giec.2017.02.009 28577773

[2] Birkmeyer JD Siewers AE Finlayson EVA Stukel TA Lucas FL Batista I. Hospital volume and surgical mortality in the United States. N Engl J Med. (2002) 346:1128–37. 10.1056/NEJMsa012337 11948273

[3] Cuschieri A Shimi S Banting S. Endoscopic oesophagectomy through a right thoracoscopic approach. J R Coll Surg Edinb. (1992) 37:7–11. 1573620

[4] Yamamoto M Weber JM Karl RC Meredith KL. Minimally invasive surgery for esophageal cancer: review of the literature and institutional experience. Cancer Control. (2013) 20:130–7. 10.1177/107327481302000206 23571703

[5] Luketich JD Schauer PR Christie NA Weigel TL Raja S Fernando HC. Minimally invasive esophagectomy. Ann Thorac Surg. (2000) 70:906–12. 10.1016/S0003-4975(00)01711-2 11016332

[6] DePaula AL Hashiba K Ferreira EA de Paula RA Grecco E. Laparoscopic transhiatal esophagectomy with esophagogastroplasty. Surg Laparosc Endosc. (1995) 5:1–5. 7735533

[7] Galvani CA Gorodner MV Moser F Jacobsen G Chretien C Espat NJ. Robotically assisted laparoscopic transhiatal esophagectomy. Surg Endosc. (2008) 22:188–95. 10.1007/s00464-007-9441-3 17939004

[8] Horgan S Berger RA Elli EF Espat NJ. Robotic-assisted minimally invasive transhiatal esophagectomy. Am Surg. (2003) 69:624–6. 12889629

[9] Prasad GA Wu TT Wigle DA Buttar NS Wongkeesong LM Dunagan KT. Endoscopic and surgical treatment of mucosal (T1a) esophageal adenocarcinoma in Barrett's esophagus. Gastroenterology. (2009) 137:815–23. 10.1053/j.gastro.2009.05.059 19524578 PMC3815672

[10] Patel V Burbridge RA. Endoscopic approaches for early-stage esophageal cancer: current options. Curr Oncol Rep. (2015) 17:421 10.1007/s11912-014-0421-1 25416315 PMC5861347

[11] Soetikno R Kaltenbach T Yeh R Gotoda T. Endoscopic mucosal resection for early cancers of the upper gastrointestinal tract. J Clin Oncol. (2005) 23:4490–98. 10.1200/JCO.2005.19.935 16002839

[12] Ning B Abdelfatah MM Othman MO. Endoscopic submucosal dissection and endoscopic mucosal resection for early stage esophageal cancer. Ann Cardiothorac Surg. (2017) 6:88–98 10.21037/acs.2017.03.15 28446997 PMC5387148

[13] Smithers BM Gotley DC McEwan D Martin I Bessell J Doyle L. Thoracoscopic mobilization of the esophagus: a 6 year experience. Surg Endosc. (2001) 15:176–82. 10.1007/s004640000307 11285963

[14] Osugi H Takemura M Higashino M Takada N Lee S Ueno M. Learning curve of videoassisted thoracoscopic esophagectomy and extensive lymphadenectomy for squamous cell cancer of the thoracic esophagus and results. Surg Endosc. (2003) 17:515–9. 10.1007/s00464-002-9075-4 12399847

[15] Kataoka K Takeuchi H Mizusawa J Ando M Tsubosa Y Koyanagi K. A randomized Phase III trial of thoracoscopic versus open esophagectomy for thoracic esophageal cancer: Japan Clinical Oncology Group Study JCOG1409. Jpn J Clin Oncol. (2016) 46:174–7. 10.1093/jjco/hyv178 26732383

[16] Manner H Pech O Heldmann Y May A Pohl J Behrens A. Efficacy, safety, and long-term results of endoscopic treatment for early stage adenocarcinoma of the esophagus with low-risk sm1 invasion. Clin Gastroenterol Hepatol. (2013) 11:630–5; quize45. 10.1016/j.cgh.2012.12.040 23357492

[17] Ahmadi A Draganov P. Endoscopic mucosal resection in the upper gastrointestinal tract. World J Gastroenterol. (2008) 14:1984–1989. 10.3748/wjg.14.1984 18395896 PMC2701517

[18] Shimizu M Zaninotto G Nagata K Graham DY Lauwers GY. Esophageal squamous cell carcinoma with special reference to its early stage. Best Pract Res Clin Gastroenterol. (2013) 27:171–86. 10.1016/j.bpg.2013.03.010 23809239

[19] Esaki M Matsumoto T Hirakawa K Nakamura S Umeno J Koga H. Risk factors for local recurrence of superficial esophageal cancer after treatment by endoscopic mucosal resection. Endoscopy. (2007) 39:41–5. 10.1055/s-2006-945143 17252459

[20] Schmidt HM1 Mohiuddin K Bodnar AM El Lakis M Kaplan S Irani S. Multidisciplinary treatment of T1a adenocarcinoma in Barrett's esophagus: contemporary comparison of endoscopic and surgical treatment in physiologically fit patients. Surg Endosc. (2015) 3391–401. 10.1007/s00464-015-4621-z 26541725

[21] Teoh AY Chiu PW Yu Ngo DK Wong SK Lau JY Ng EK. Outcomes of endoscopic submucosal dissection versus endoscopic mucosal resection in management of superficial squamous esophageal neoplasms outside Japan. J Clin Gastroenterol. (2010) 44:e190–4. 10.1097/MCG.0b013e3181ce52fb 20844363

[22] Terheggen G Horn EM Vieth M Gabbert H Enderle M Neugebauer A. A randomized trial of endoscopic submucosal dissection versus endoscopic mucosal resection for early Barrett's neoplasia. Gut. (2017) 66:783–93. 10.1136/gutjnl-2015-310126 26801885 PMC5531224

[23] Kvasha A Kvasha V Hadary A Willenz U Szvalb S Waksman I. Endoluminal rectal resection and transanal natural orifice specimen extraction (NOSE) without rectal stump opening: a novel, clean surgical technique in a porcine model. Surg Innov. (2013) 20:454–8. 10.1177/1553350612468509 23222059

[24] Saida Y Nagao J Nakamura Y Nakamura Y Enomoto T Katagiri M. A comparison of abdominal cavity bacterial contamination of laparoscopy and laparotomy for colorectal cancers. Dig Surg. (2008) 25:198–201. 10.1159/000140689 18577864

[25] Kvasha A Rosenthal E Khalifa M Waksman I. Notes assisted endolumenal rectal resection and specimen extraction without rectal stump opening - our experience with this novel technique in a porcine model. Harefuah. (2017) 156:307–10. 28551914

